# Ex vivo comparative study of at home bleaching products on whitening efficacy and enamel integrity

**DOI:** 10.1038/s41598-025-27442-7

**Published:** 2025-12-10

**Authors:** Claudio Pasquale, Fabrizio Barberis, Alberto Lagazzo, Luca Ceseracciu, Matteo Schiaffino, Stefano Benedicenti, Giovanni Lo Bello, Cecilia Beccari, Roberto Raiteri, Andrea Amaroli

**Affiliations:** 1 Department of Civil, Chemical and Environmental Engineering, University of Genoa, 16100 Genoa, Italy; 2 Materials Characterization Facility, Istituto Italiano di Tecnologia (IIT), 16163 Genoa, Italy; 3 Dipartimento di Scienze Chirurgiche e Diagnostiche Integrate, University of Genoa, 16132 Genoa, Italy; 4 Department of Informatics, Bioengineering, Robotics and System Engineering, University of Genoa, 16145 Genoa, Italy; 5 BIO-Photonics Overarching Research Laboratory, Department of Earth, Environmental and Life Sciences, University of Genoa, Viale Benedetto XV, 5, 16132 Genoa, Italy; 6 Interuniversity Center for the Promotion of the 3Rs Principles in Teaching and Research (Centro 3R), 56122 Pisa, Italy

**Keywords:** Home-based bleaching, Carbamide peroxide, Esthetic dentistry, Bleaching, Whitening, Hydrogen peroxide, Gingival irritation, Enamel erosion, Tooth discoloration, Demineralization, Dentistry, Dental conditions, Dental materials, Dental treatments, Special care dentistry

## Abstract

To compare the efficacy and safety of at-home bleaching products on dental enamel using an ex vivo bovine enamel model. The study aimed to evaluate the whitening effectiveness and the structural impact of these treatments on enamel integrity. Four at-home bleaching agents [BlancOne Home Night + (BlancOne; 12% carbamide peroxide/4.2% hydrogen peroxide + nano-hydroxyapatite), Opalescence PF Regular (Opale; 16%/5.7% + potassium and fluoride nitrate), Zoom Nite White (Zoom; 16%/5.7% + amorphous calcium phosphate), and Pola Night (Pola; 16%/5.7% + potassium fluoride)] were tested on bovine enamel samples according to ISO 28,399:2021 standards. Whitening efficacy was measured spectrophotometrically before and 48 h after treatments. Enamel surface changes in topography and mechanical properties were analyzed using Atomic Force Microscopy (AFM) and nanoindentation. The pH values of the agents were monitored throughout the treatment to assess their acidity. All agents effectively whitened enamel, with BlancOne demonstrating greater whitening efficacy than Zoom (ΔOD: 5.7 ± 1.1 vs. 3.7 ± 0.9; *p* < 0.001) and Pola (ΔOD: 5.6 ± 1.7 vs. 1.8 ± 1.0; *p* < 0.001). No significant difference was observed between BlancOne and Opale (ΔOD: 5.1 ± 0.9 vs. 4.4 ± 1.9; *p* > 0.05). AFM and nanoindentation analyses revealed significant differences in the integrity of the enamel surface. BlancOne, Zoom and Pola induced only minimal increases in surface roughness and reductions in hardness, whereas Opale resulted in the most pronounced enamel alterations [Control = 4.75 (× 1000 GPa) ± 1.57; BlancOne = 3.1 ± 1.4; Opale = 0.1 ± 0.03; Zoom = 2.9 ± 1.0; Pola = 3.2 ± 0.9. Control vs BlancOne, Zoom, and Pola: *p* < 0.05; Control vs Opale: *p* < 0.001]. The pH analysis indicated that BlancOne and Opalescence had less acidic profiles (pH 6–7) than Zoom and Pola (pH ≈ 5.5). This study demonstrates that although all tested agents effectively achieve whitening, they differ in consistency and impact on enamel integrity. Among the tested agents, the formulation containing 12% carbamide peroxide (equivalent to 4.2% hydrogen peroxide) and nano-hydroxyapatite demonstrated a favorable balance between bleaching efficacy and enamel structure preservation. These findings provide valuable insights for clinicians in selecting whitening protocols that minimize enamel damage while achieving satisfactory cosmetic outcomes.

## Introduction

The concept of a perfect, bright white smile is deeply rooted in modern culture, shaped by a complex interplay of historical, cultural, social, and economic factors that collectively influence perceptions of beauty and self-image^1^. In recent years, tooth whitening has become one of the most sought-after cosmetic treatments, reflecting the increasing interest among patients not only in improving the aesthetics of their smiles but also in enhancing self-confidence and psychosocial well-being^2^. This desire, fuelled by media and the growing emphasis on personal aesthetics, has led to the widespread adoption of whitening products for both professional and at-home use^3^. However, while these products can improve patients’ appearance and boost their confidence, it is essential to consider their long-term effects on dental health^4^.

The whitening process relies on oxidizing agents, such as hydrogen peroxide (HP) and carbamide peroxide (CP), which break down the chromogenic molecules trapped within dental enamel^5,6^. In professional treatments, high concentrations of HP (up to 40%) enable faster and more effective whitening but require careful supervision to minimize the risk of irritation and sensitivity^7^. Conversely, at-home treatments typically use lower peroxide concentrations, ranging from 3 to 10%, to reduce side effects; however, this compromise results in a more extended treatment duration to achieve visible results, leading many patients to favor professional options^7,8^. The literature suggests that whitening treatments can have adverse effects if misused or used without supervision. For instance, the indiscriminate use of whitening strips has been associated with increased tooth sensitivity, a common side effect of whitening treatments^9^. Additionally, the application of peroxides can cause gum irritation, manifesting as inflammation or discomfort^10^. Furthermore, peroxides can have adverse effects on restorative dental materials^11^, compromising their integrity and durability. The combined use of whitening toothpaste and professional whitening treatments can also increase enamel roughness, facilitating biofilm accumulation and thereby increasing the risk of caries and periodontal diseases^12^. Excessive use of whitening products can also lead to microstructural changes in enamel, making it more susceptible to damage and staining^13–15^. New formulations and technologies have been developed in response to the growing demand for less invasive yet effective whitening treatments, including LED photoactivation in professional treatments. Photoactivated treatments, such as the HP system with specific wavelength light used in BlancOne ULTRA +, demonstrate an improved balance of efficacy and safety compared to non-photoactivated products^16^. A recent review on dental whitening found that light-activating sources, such as lasers or LEDs, were associated with enhanced whitening efficacy^17^.

However, the cost-effectiveness of whitening treatments remains debated, as outcomes can vary with the product and practitioner^18,19^. Building on prior findings from professional treatments^16^, this study compares the efficacy and safety of at-home whitening products, aiming to balance aesthetics with oral health.

The predictor variable was the innovative formulation of the BlancOne Home Night + (Blanc) treatment, which uses 12% CP and nano-hydroxyapatite, as described on the company website (www.blancone.eu). The study’s primary endpoint was to assess whether the newly launched Blanc outperforms other at-home teeth-whitening products in terms of effectiveness. The study’s secondary endpoint was to investigate potential adverse effects of bleaching processes on enamel. The null hypothesis was that no significant difference in whitening efficacy or enamel safety would be observed between Blanc and the other tested treatments.

## Materials and methods

### Agents for home whitening

The whitening agents assessed in this comparative study (Table 1) included three widely available at-home products and a newly launched one.

**Table 1:** Brief description of bleaching products, including the number and time of applications.

Agents name	% CP	Additives for enamel preservation	Applications	Application times
Opalescence PF regular	16% (5.7%HP)	Potassium nitrate	10–15	All night
		Potassium fluoride		
Zoom nite white	16% (5.7%HP)	ACP	10–15	All night
		Potassium nitrate		
		Potassium fluoride		
Pola night	16% (5.7%HP)	Potassium fluoride	10–15	All night
BlancOne Home night +	12% (4.2%HP)	0.5% nHAP	10–15	All night

CP, carbamide peroxide; HP, hydrogen peroxide; nHAP, nano-hydroxyapatite; ACP, amorphous calcium phosphate.

Opalescence PF Regular with 16% CP (Opale) (ULTRADENT, 505 West Ultradent Drive, South Jordan, UT 84095, USA);

Zoom Nite White with 16% CP (Zoom) (Philips Research Eindhoven, High Tech Campus 34, 5656 AE, Eindhoven, The Netherlands);

Pola Night with 16% CP (Pola) (SDI Limited, 3–15 Brunsdon Street, Bayswater, Victoria 3153, Australia);

BlancOne Home Night + with 12% CP (Blanc) (IDS SpA, Via Valletta San Cristoforo, 28/10, 17100 Savona, Italy).

### Specimens’ collection and preparation

#### Recruitment of bovine mandibles

Bovine samples were selected in line with a previous work^16^. According to the literature^20,21^, it is considered a reliable ex vivo material for pre-clinical screening studies, as validated by ISO 28,399:2021 standards for external tooth bleaching tests^22^. Specifically, 18 bovine mandibles (six for each comparative experiment) were obtained from Azienda Agricola BIOBIO’s slaughterhouse in Vobarno, Brescia, Italy. The specimens, 18 months old, were bred for human consumption in accordance with Italian Ministry of Agriculture, Food, and Forestry regulations. Since the animals were not bred or sacrificed at the University of Genoa for research purposes, ethics committee approval was not required following current ethical legislation.

To minimize tooth-to-tooth variability, specimens were bred on the same organic farm, fed the same diet, and never subjected to antibiotic treatment until slaughter. The specimens were collected and processed immediately after slaughter, complying with safety regulations. The incisors were kept intact within the mandibles, preserving the skeletal portions, and were cleaned using standard dental prophylaxis. 144 incisors were treated, 8 in each jaw (four on the left and four on the right).

#### Preparation of bovine mandibles for experimental treatments

**Fig. 1:**
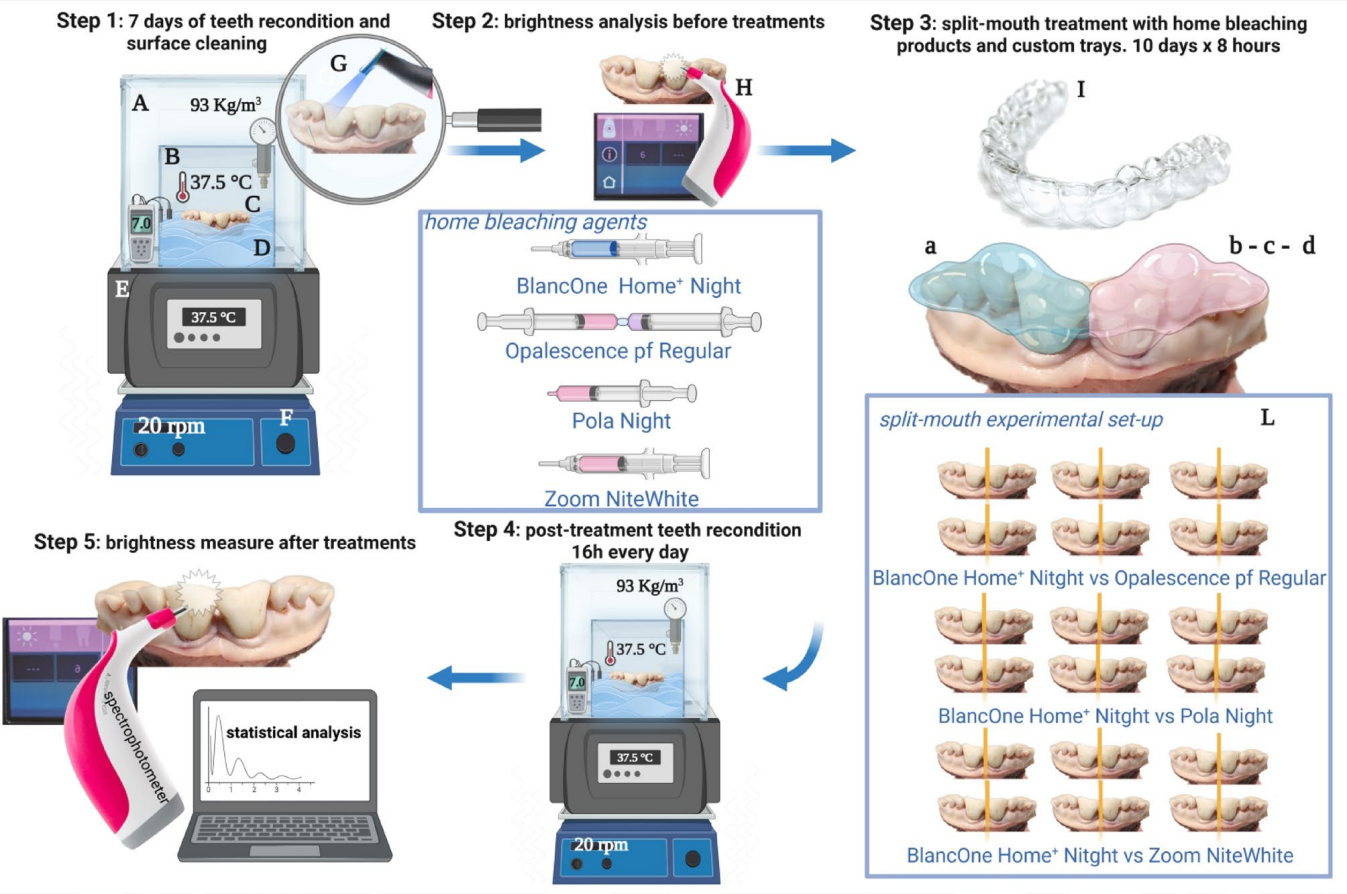
Experimental design. Step 1: Bovine mandibles were pre-incubated in a bioreactor designed and characterized by us^16^ for 7 days before the experiments took place to re-condition the teeth. The oral cavity simulator bioreactor consists of a thermostatic bath (A), filled with distilled water. The bath contains a cubic glass tank (B) immersed. The mandible is fixed to a stub support on the tank bottom (C). Salivary substitute solution (D) is maintained at 37.5 °C in a thermostatic bath (E) and shaken at 20 rpm (F) to allow the oscillating artificial saliva to contact the tooth surfaces of the samples. The pH (7.0), temperature (37.5 °C), and humidity (93 kg/m^3^) are monitored through a pH Meter, a thermocouple, and a digital thermo-hygrometer, respectively. Before treatment, the tooth surface is cleaned with an airflow, erythritol powder, and water (G). Step 2: The brightness is analyzed with a special spectrophotometer (H) before the treatment. The bleaching is performed through (a) Blanc and (b) Opale, (c) Pola, or (d) Zoom. Step 3: Custom trays (I) were created for all mandibles holding home-bleaching products. Tests are performed through a split-mouth experimental set-up, where 18 mandibles are analyzed, six mandibles for each comparative experiment (L). The treatments were performed over 10 days, each lasting 8 h (the average time for an overnight exposure). Step 4: Post-reconditioning was achieved by keeping the mandibles in the bioreactor during non-whitening treatment hours. Step 5: The brightness is measured with the spectrophotometer 48 h after the last treatment.

A brief description of the experimental setup is shown in Fig. 1. The procedure followed the ISO 28,399:2021 standards^22^.

Before proceeding with the treatments, to avoid altering the characteristics of the tooth surfaces, the mandibles were kept in a unique bioreactor for 7 days, which simulates oral conditions. The bioreactor reproduces a human-like oral condition, as was extensively described in previous studies^16,23^. In summary, we used a customised bioreactor capable of replicating the interaction between salivary fluid and tooth surface to simulate human oral conditions through precise flow control (Fig. 1, step 1). The oral environment was simulated using artificial saliva at a neutral pH, monitored with a Five Easy Plus pH Meter (FP20-Std-Kit: Mettler Toledo, Columbus, Ohio, USA). The temperature was maintained at 37.5 °C and humidity at 93 kg/m^3^, through specific thermal (Hanna Instruments Italia srl—Viale Delle Industrie, 11-35010 Ronchi di Vil-lafranca Padovana, Italy) and humidity (Hanna Instruments Italia srl) devices (Fig. 1, step 1A,B,C,D,E). An orbital oscillator (SLA-OS-200, Scitech LabApp Ltd, Micklefield, Leeds, LS25 9BP, UK) was used to simulate continuous contact of saliva with the tooth surface (Fig. 1, step 1F).

A surface cleaning was performed using the Electro Medical Systems air flow technique (E.M.S. Electro Medical Systems S.A., Chem. de la Vuarpillière 31, 1260 Nyon, Switzerland) with erythritol powder and water^24^ (Fig. 1, step 1G).

**Fig. 2:**
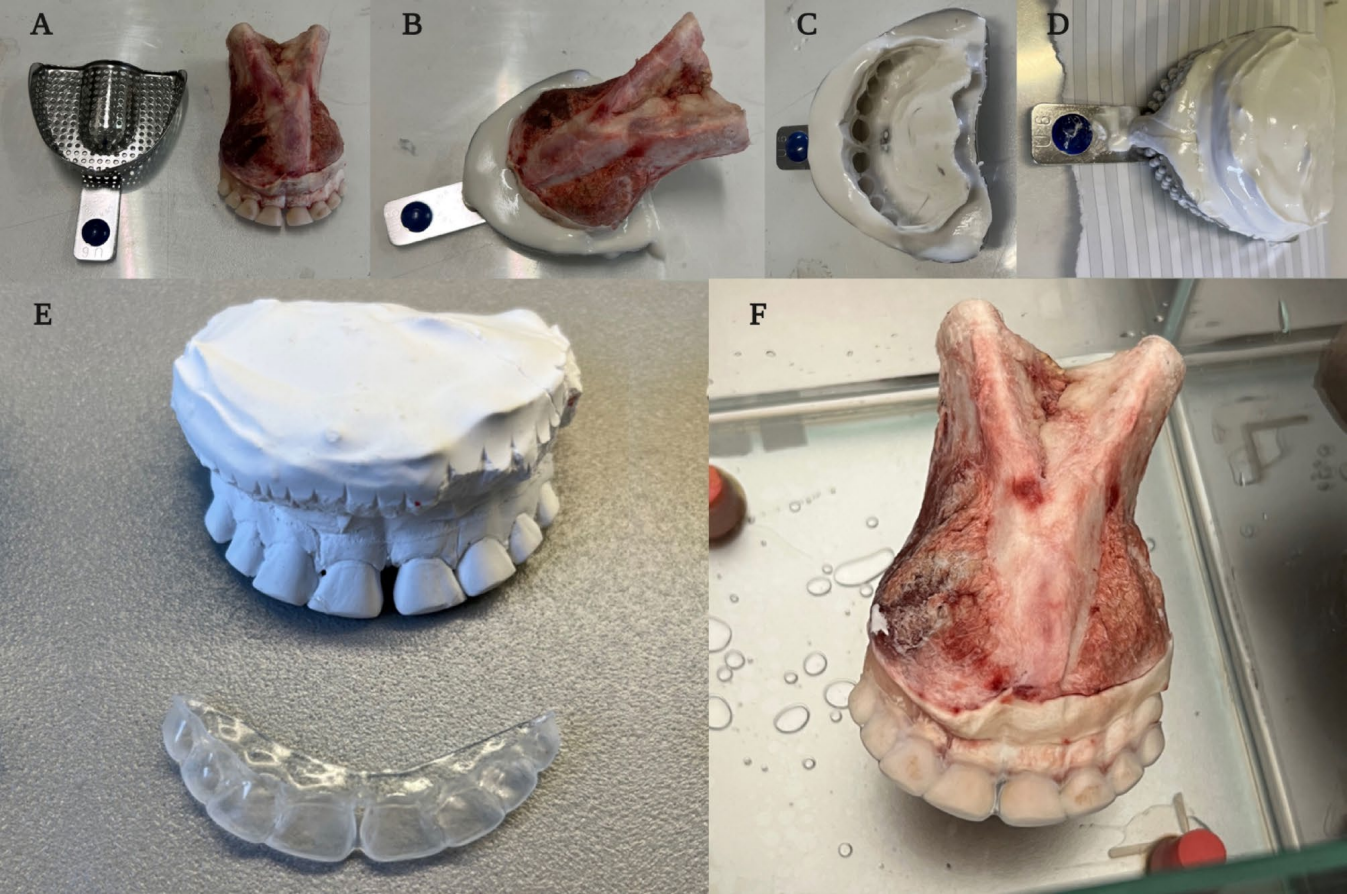
Description of the procedure to create newly manufactured tray whiteners for bovine teeth. (**A**) Mandible and impression tray; (**B**) Bovine dental impression; (**C**) Negative impression in alginate agar–agar; (**D**) Developing of plaster model; (**E**) Plaster model and customized mask; (**F**) Customized mask on bovine mandible teeth.

The home bleaching treatment involves the application of the product inside standard whitening trays supplied by the whitening product manufacturer or customised and built on the patient’s dental moulds. They are made of thermoplastic material and thermoformed using a device that operates under vacuum technology. Since they are not anatomically usable for the teeth of bovine jaws, we have fabricated whitening trays from scratch (Fig. 1I and Fig. 2). Therefore, as described in Fig. 2, dental impressions were taken with agar–agar alginate for each bovine mandible subjected to bleaching treatment. Corresponding models were developed using type II plaster, on which the bleaching trays used in split-mouth treatments were subsequently fabricated.

### Spectrophotometer evaluation of bovine mandible teeth bleaching

The tests were conducted by working on one mandible side at a time (Fig. 1a-b-c-d, step 3).

The treatment on the right and left mandibular incisors was randomly allocated (1:1) to be treated with either Blanc or classical market products for home use, such as Opale, Zoom, or Pola. Randomization was based on a random-sequence software program (www.random.org/sequences), which generates randomised sequences.

The brightness was analysed before treatments using the spectrophotometer, VITA easy-shade (VI-TA Zahnfabrik (VITA Zahnfabrik H. Rauter GmbH & Co. KG Postfach 1338 D-79704 Bad Säckingen) (Fig. 1 H, step 2). The bleaching treatment involved applying the at-home tooth whitening products described in the previous sections. The application was carried out using the whitening trays we produced, following the manufacturer’s product delivery procedures outlined in Table 1. Then, the measure was performed with the exact spectrophotometer 48 h after the bleaching treatment (Fig. 1, step 4). As per the literature, the measurements were taken 48 h after treatment to confirm the final result and treatment stability^25^.

48 h after the spectrophotometer measurement was performed (Fig. 1, step 5). The effectiveness of bleaching values was expressed as optical density (OD)^26^. The average effectiveness is expressed as ΔOD, i.e., the difference in OD measured spectrophotometrically after treatment minus the OD measured before treatment. The operators who performed the treatment, those who performed the spectrophotometer measurements, and those who performed the data analysis were blind to each other. To assess the whitening effect, a brightness-based scale (“VITA Bleached Scale”) was adopted, which relies on a measurement system that excludes the chromatic components of colors perceivable by the human eye or photographic devices. Specifically, the positive and negative contributions of the green/red and blue/yellow chromatic axes (ranging from − 128 to + 128) are not considered, as they can introduce variability in color perception. They may compromise the objective evaluation of whitening efficacy.

The adopted method focuses exclusively on brightness, interpreted as an index of light reflection in a grayscale spectrum: 0 corresponds to absolute white, while − 100 corresponds to absolute black. This approach ensures that measurements are independent of the sample’s residual chromatic hue, thereby minimizing distortions associated with colorimetric components.

This methodological choice aligns with recent literature, which recommends overcoming the limitations of traditional colorimetric scales in dental bleaching by enabling the collection of more linear and statistically manageable numerical data^27,28^.

However, although the literature highlights the difficulty of comparing experimental setups due to the variability in treatment efficacy as a function of initial tooth color—since darker baseline shades tend to exhibit greater changes than lighter ones, regardless of the technique or product used^29,30^—the samples were also evaluated using the conventional “VITA 3D-MASTER Guide”. This was done to allow broader comparability with existing data and to maintain a recognized clinical reference. The VITA Bleached Guide is an extension of the VITA 3D-Master Shade Guide, specifically developed to include lighter shades (such as 0M1 and 0.5M1) for evaluating tooth whitening outcomes.

The 3D-Master system is based on three fundamental parameters: Value: indicates the brightness or lightness of the tooth; Chroma: refers to the intensity or saturation of the color; Hue: represents the color shade, reflecting its tendency toward yellowish or reddish tones.

Each tooth shade is classified using the xMy notation, where:

x = a number from 1 to 5, representing the value (1 = lightest, 5 = darkest).

M = medium hue.

y = a number from 1 to 3, indicating the chroma (1 = less saturated, 3 = more saturated).

### Structural and mechanical analyses of the bovine mandibles teeth bleaching treated

Mechanical and structural analyses were conducted on 8 × 8 mm enamel sections from the central incisors of bovine mandibles treated for bleaching, as described in Fig. 1 and Section “Spectrophotometer evaluation of bovine mandible teeth bleaching”. Considering the experience from a previous work^16^, we aimed to enable a comparative structural analysis at the nanoscale and to assess the heterogeneity of the surface structure across different regions of the same sample. To achieve this, we selected the same 20 × 20 µm^2^ areas for comparison before and after bleaching treatment.

For this purpose, the areas of interest were manually demarcated by surface incision with a dental drill. The areas were thus easily identifiable by optical inspection, facilitating the repositioning of the atomic force microscope probe over the same scanning area.

#### Atomic lpy (AFM) analyses of the bovine mandible teeth bleaching treated

To obtain precise measurements of enamel surface topography height before and after bleaching treatment, we employed correlative atomic force microscopy using a commercial AFM (NanoWizard 4 XP BioScience, Bruker) equipped with a motorized stage for precise sample repositioning (HybridStageTM, Bruker) and integrated with an upright optical microscope (AxioZoom v.16, Zeiss). On each sample, a few small features, visible under the optical microscope, were manually marked to create position markers. In addition, using the “direct overlay” and “optical tiling” features of the AFM software, it was possible to select the scan area directly onto the optical image with an accuracy of a few micrometers. This allowed us to position the AFM probe over the tooth surface with sufficient accuracy to scan the same 20 × 20 µm^2^ area before and after the bleaching treatment.

Topographic images were acquired in ‘contact mode’ with the sample immersed in deionized water. To maintain consistency of the results, the same probe was used for all measurements (HQ/Hard/Al BS, MikroMasch with a spring constant k = 0.255 N/m and a resonant frequency f0 = 13 kHz), which is characterized by a wear-resistant coating that ensures a constant tip geometry and size over multiple scans. The measurements were performed at a scan rate of 0.4 Hz with a resolution of 512 × 512 pixels. This allowed quantitatively comparable topographic characteristics to be obtained for the different samples before and after bleaching treatment.

AFM data analysis was performed with Mountains® software (Digital Surf, France). The Developed Interfacial Area Ratio (Sdr) parameter was calculated according to ISO 25178-2:2012 to quantify and compare surface roughness variations. The Sdr parameter expresses the percentage of additional area generated by topography compared to an ideal plane of the same size as the scanned area. For a flat surface, Sdr corresponds to 0%, increasing with the complexity of the surface topography^31^.

#### Nanoindentation analyses of the bovine mandibles teeth bleaching treated

Nanoindentation measurements were performed to assess changes in the mechanical properties of enamel following bleaching treatment. Precisely, Young’s modulus, which describes the elasticity or stiffness of the material, and hardness, which represents the resistance to indentation, were calculated using the widely accepted Oliver and Pharr method^32^.

$$Young^{\prime}s\;modulus = \frac{dP}{{dh}}\frac{1}{2}\frac{{\sqrt {pgreco} }}{\sqrt A }$$

$$Hardness = \frac{P}{A}$$

In detail, P represents the load, A represents the contact area, and h represents the penetration depth. The three-sided Berkovich indenter, the most commonly used geometry for nanoindentation tests, was employed, and a maximum applied load of 5 mN corresponded to a penetration depth in the range of 200–600 nm.

Thirty measurements were thus obtained, and the results were expressed as mean ± standard deviation. The UNHT3 | NST3 (Step 700 NC) instrument from Anton Paar Italia S.r.l. (10098 Rivoli, Italy) was used for these experiments.

### Evaluation of pH of bleaching agent before and after the bovine mandible teeth treatments

The pH changes during the bleaching treatment were analysed using the pH Meter Five Easy Plus FP20-Std-Kit (Mettler Toledo, Columbus, OH, USA). The dynamic pH of the bleaching products listed in Table 1 was measured by comparing the initial pH value (pH T0) with the pH value at 4 h post-treatment (pH T4) and after “all night” (8 h) at the end of the treatment (pH T8). The evaluation times were determined according to the manufacturers’ instructions for each bleaching product.

### Statistical analysis

The statistical analysis was performed using MATLAB (The MathWorks, Inc., 1 Apple Hill Drive, Natick, Massachusetts, 01760 USA). Means ± standard deviations or differences between means (pre-treatment and post-treatment) ± standard deviations were calculated and compared.

Data distribution was assessed using the Shapiro–Wilk test, followed by statistical analysis conducted via ANOVA with Tukey’s post hoc test or Student’s t-test, as appropriate.

The ClinCalc Sample Size Calculator determined the sample size using Rosner’s formulas^33^. From preliminary data and our previous study^16^ on the primary endpoint (mean ΔOD efficacy, defined as the difference between spectrophotometric measurements pre- and post-treatment), an average ΔOD of 7.1 with a standard deviation of 30% in group 1 (Blanc) and a 15% difference in group 2 (standard commercial products) were estimated. The required sample size ranged between 118 and 194 total teeth to achieve statistical power between 0.8 and 0.95, with a significance level of α = 0.05 and a β error of 0.2.

Statistical power was calculated using an allocation ratio 1:1 and a significance level of α = 5% using the Simple Interactive Statistical Analysis (SISA) online tool, available at https://www.quantitativeskills.com/sisa/index.htm.

## Results

### Results of AFM analysis on bleached bovine mandibular teeth

**Fig. 3:**
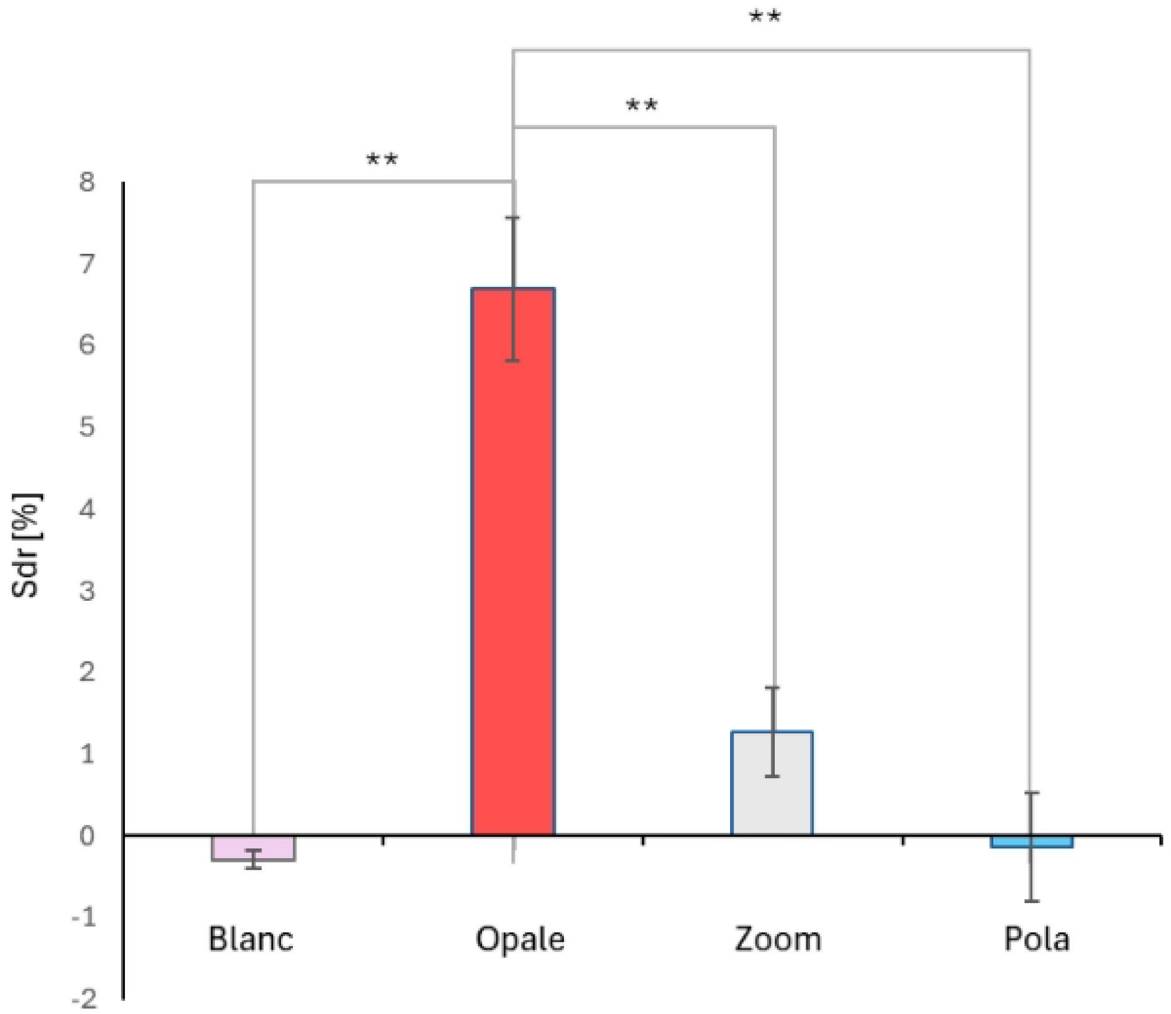
AFM analysis of bleached bovine mandibular teeth. Differences in the developed surface area (Sdr) parameter, calculated from AFM topography images before and after different at-home bleaching treatments. The x-axis (abscissa) labels represent the treatment groups. The bars display the mean ± standard error (*n* = 10). The symbol ** indicates a highly significant difference (*p* < 0.001).

The graph in Fig. 3 represents the differences in the Sdr parameter, which measures surface roughness. Sdr values, expressed as a percentage (%), were calculated from the topography image before and after the different bleaching treatments.

The Bleached Bovine Mandibular Teeth analysis shows that Blanc and Pola did not induce any significant change in roughness, indicating a minimal impact on the enamel surface. Zoom caused an increase in roughness, although less significant compared to Opale. The latter exhibited the highest growth in roughness, suggesting a more pronounced abrasive or demineralizing effect, which could compromise the integrity of the enamel.

### Results of nanoindentation analysis on c

**Fig. 4:**
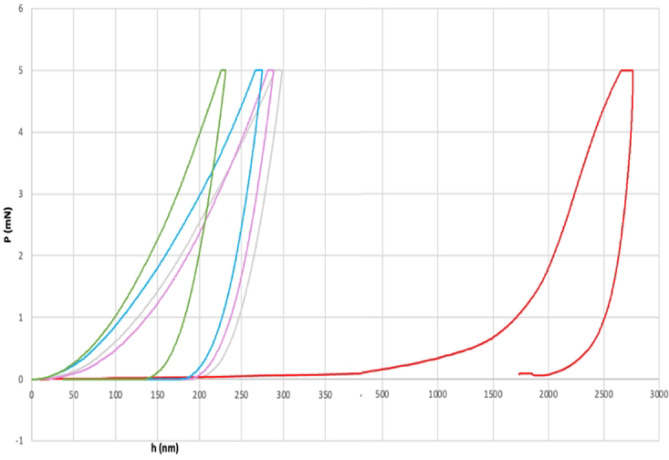
Nanoindentation analysis. Total penetration depth at the maximum loading force. Coloured lines indicate: Control = green; Pola = blue; Blanc = purple; Zoom = grey; Opale = red.

Figure 4 illustrates the total penetration depth at the maximum loading force. Samples treated with Opale exhibit a ten-fold increase in penetration depth under the same applied load of 5 mN compared to the untreated sample (control) and the other treatments such as Blanc, Zoom or Pola.

**Fig. 5:**
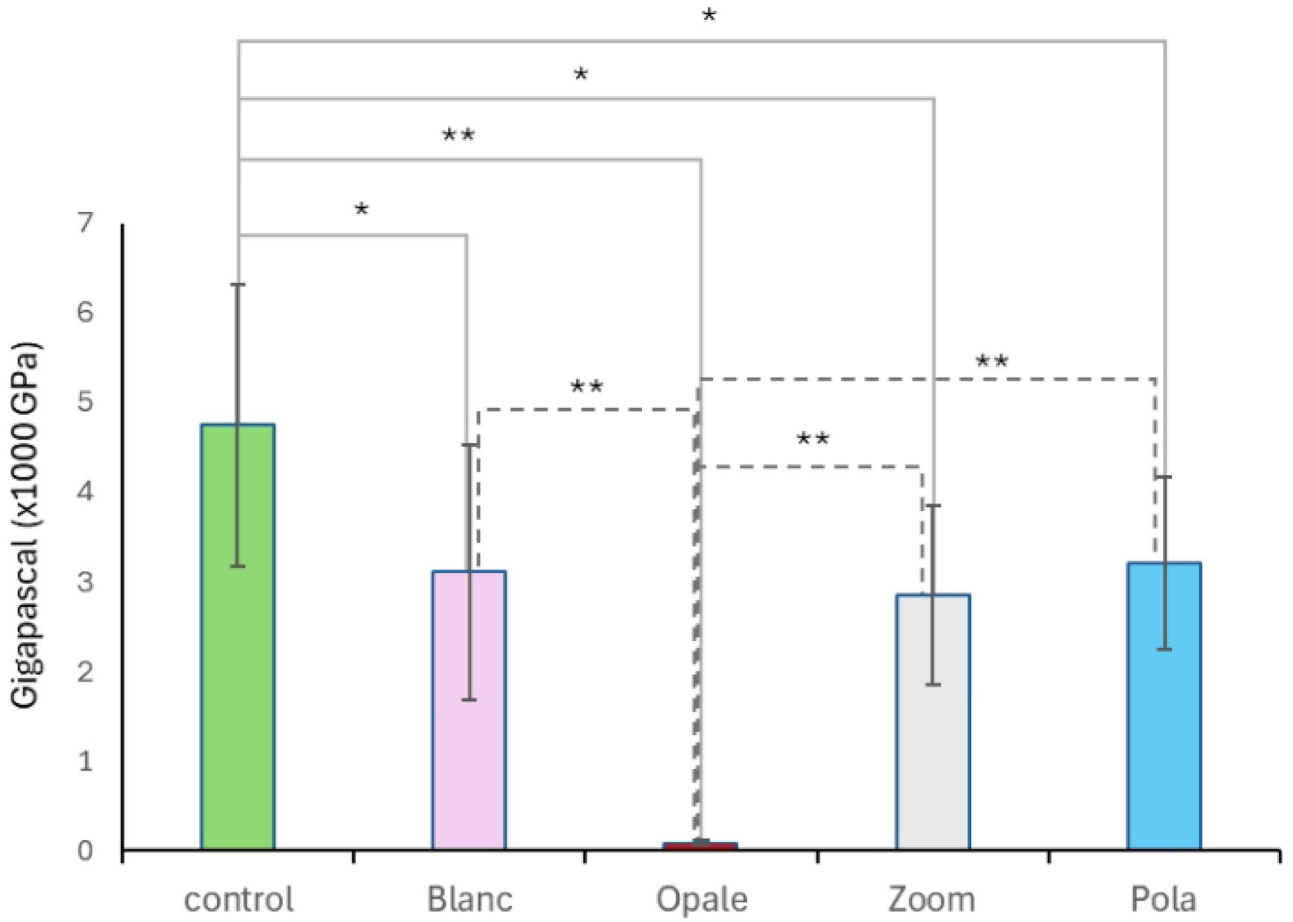
Hardness changes analysis. The graph represents the variations in enamel hardness in bleached bovine mandibular teeth. The symbols ** and * indicate an extremely statistically significant difference (*P* < 0.0001) and a statistically significant difference (*P* < 0.05), respectively, calculated using a one-way ANOVA test followed by the Tukey HSD test (*n* = 10). The x-axis labels represent the treatment groups.

The hardness changes shown in Fig. 5 highlight that, compared to the control, Opale exhibited a significantly lower value, indicating a marked reduction in hardness (*P* < 0.0001). This reduction is also highly statistically significant between Opale and the other treatments (*P* < 0.0001). In contrast, the variation in hardness after treatments with Zoom, Pola, and Blanc is less evident than in the control, although the reduction remains statistically significant (*P* < 0.05).

**Fig. 6:**
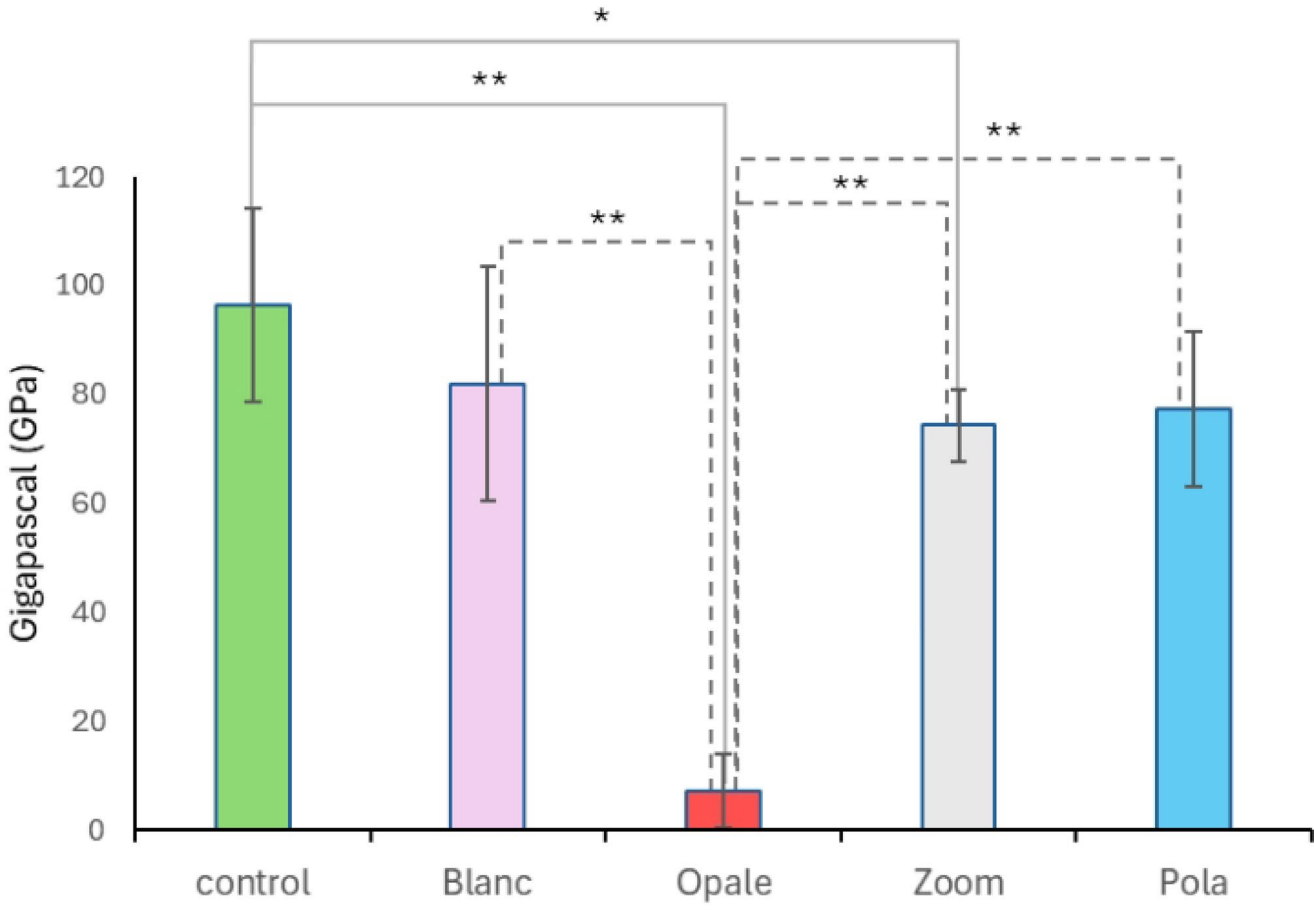
Young’s modulus analysis. The graph represents the variations in brittleness and elasticity of bleached bovine mandibular teeth. The symbols ** and * indicate an extremely statistically significant difference (*P* < 0.0001) and a statistically significant difference (*P* < 0.05), respectively, calculated using a one-way ANOVA test followed by the Tukey HSD test (*n* = 10). The x-axis labels represent the treatment groups.

Regarding Young’s modulus, Fig. 6 illustrates the effect of all treatments on this parameter. In particular, Opale shows the most pronounced effect, indicating a tooth that is more brittle and less elastic compared to the control (*P* < 0.001) and the other treatments (*P* < 0.001). Zoom also shows a significant alteration in Young’s modulus, although it is much less evident compared to Opale (*P* < 0.05).

### Results of spectrophotometric analysis on bleached bovine mandibular teeth

**Fig. 7:**
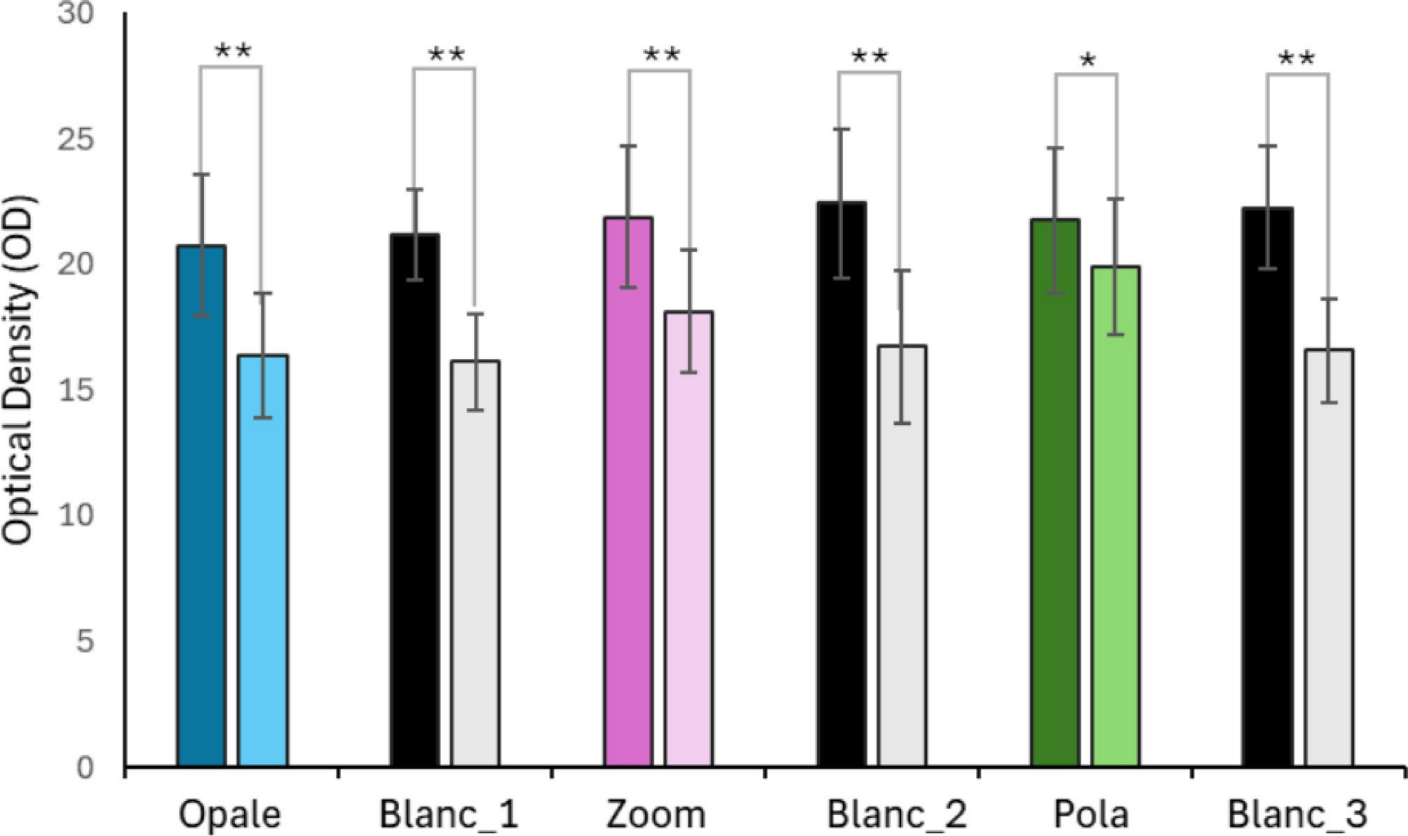
Effect of at-home bleaching treatments. The histograms show the means ± standard deviations of spectrophotometric brightness measurements, expressed as optical density (O.D.), taken before (serving as control values) and 48 h after the treatments. The adopted method focuses exclusively on brightness, interpreted as an index of light reflection in a grayscale spectrum: 0 corresponds to absolute white, while − 100 corresponds to absolute black. The symbols * and ** indicate, respectively, a significant difference (*p* < 0.05) and a highly significant difference (*p* < 0.0001) according to the Student’s t-test (*n* = 24), comparing tooth brightness before (left column with more intense colour) and after (right column with paler colour) bleaching treatments. The x-axis labels represent the treatment groups.

Using a spectrophotometer allowed for the evaluation of tooth brightness before and after bleaching treatments. Brightness data are expressed as the decimal logarithm of the ratio between the intensity of incident light and the intensity of transmitted light, referred to as optical density (O.D.). The results are graphically presented in Fig. 7.

Statistical analysis was performed using the Student’s t-test and the mean O.D. (*n* = 24). Values before treatment were compared with those measured 48 h post-treatment. The analysis revealed that the Blanc treatment positively improved tooth brightness across all three experiments, with a highly significant effect (*p* < 0.0001). Similarly, treatments with Opale and Zoom demonstrated a highly significant positive impact on tooth brightness (*p* < 0.0001). The Pola treatment also produced a statistically significant improvement, albeit to a lesser degree (*p* < 0.05). The ANOVA analysis, followed by HSD Tukey’s test, confirmed the uniformity of the initial samples, showing no significant differences in brightness among the means of the six starting conditions (left columns with more intense colour).

The evaluation of whitening treatment efficacy using the 3D-Master system confirms the spectrophotometric brightness assessment results (Table 2).

**Table 2:** Evaluation of whitening treatment efficacy using the 3D-Master system.

Treatment	3D-master classification before	3D-master classification after	Efficacy
Blanc	4.5M2/4M2	3M2/2.5M2	●●●
Opale	4.5M2/4M2	3M2/2.5M2	●●●
Zoom	4.5M2/4M2	3.5M2/3M2	●●
Pola	4.5M2/4M2	4M2/3.5M2	●

The system is based on three fundamental parameters: tooth brightness or lightness, color intensity or saturation, and color shade. Each tooth shade is classified using the xMy notation, where x = a number from 1 to 5, representing the value (1 = lightest, 5 = darkest); M = medium hue; y = a number from 1 to 3, indicating the chroma (1 = less saturated, 3 = more saturated). ●●● = 6-place improvement on the 3D-Master system scale; ●● = 4-place improvement on the scale; ● = 2-place improvement on the scale.

### Result of whitening effects comparison evaluated by spectrophotometric analysis of bleached bovine mandibular teeth

**Fig. 8:**
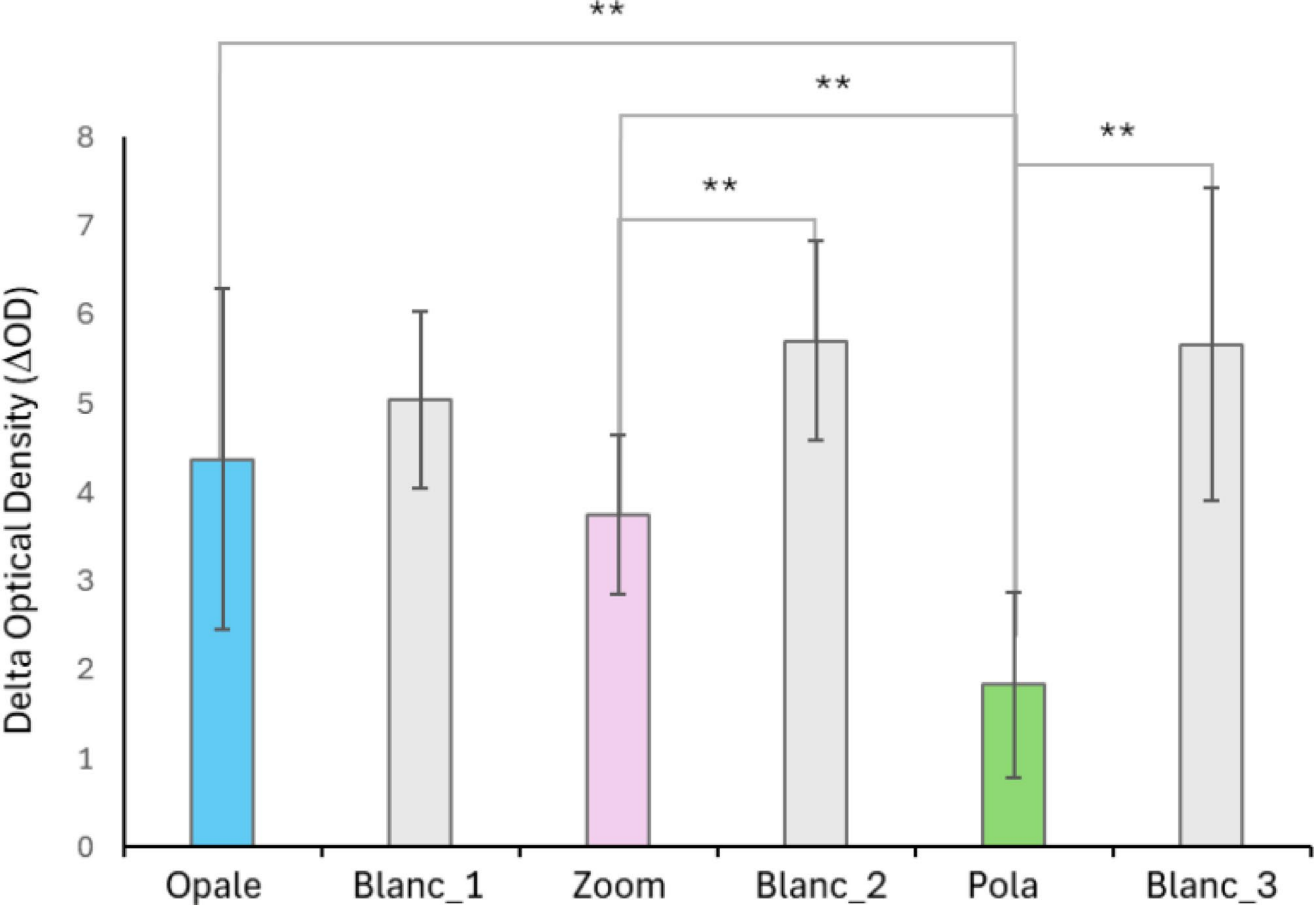
Effect of at-home bleaching treatments. The histograms display the means ± standard deviations of spectrophotometric brightness measurements, calculated as the difference between the pre-treatment values and those recorded 48 h post-treatment (delta optical density, ΔO.D.). The symbol ** denotes a highly significant difference (*p* < 0.0001) as determined by ANOVA followed by Tukey’s HSD test (*n* = 24). The x-axis labels indicate the treatment groups. _1, 2, and 3 correspond to the Blanc treatment applied in the experiments comparing Opale, Zoom, and Pola treatments, respectively.

A comparative analysis of the whitening effects of the various treatments was performed to highlight any qualitative differences. For this purpose, an analysis of variance (ANOVA) followed by HSD Tukey’s test was conducted (*n* = 24). The change in optical density (ΔOD), or the difference between pre-treatment measurements and those taken 48 h post-treatment, was calculated to assess the effect of the treatments. The results are presented in Fig. 8. The comparison revealed that the difference between the effect of the Blanc treatment and those of Zoom and Pola was highly significant (*p* < 0.0001). The effect of the Opale treatment on brightness was similar to that of Blanc (*p* > 0.05).

Furthermore, a highly significant statistical difference was observed between the Opale and Zoom treatments compared to the Pola treatment (*p* < 0.0001). No statistically significant difference was found between the effects of Opale and Zoom (*p* > 0.05).

### Result of pH comparison of bleaching agents used on bovine mandibular teeth

**Fig. 9:**
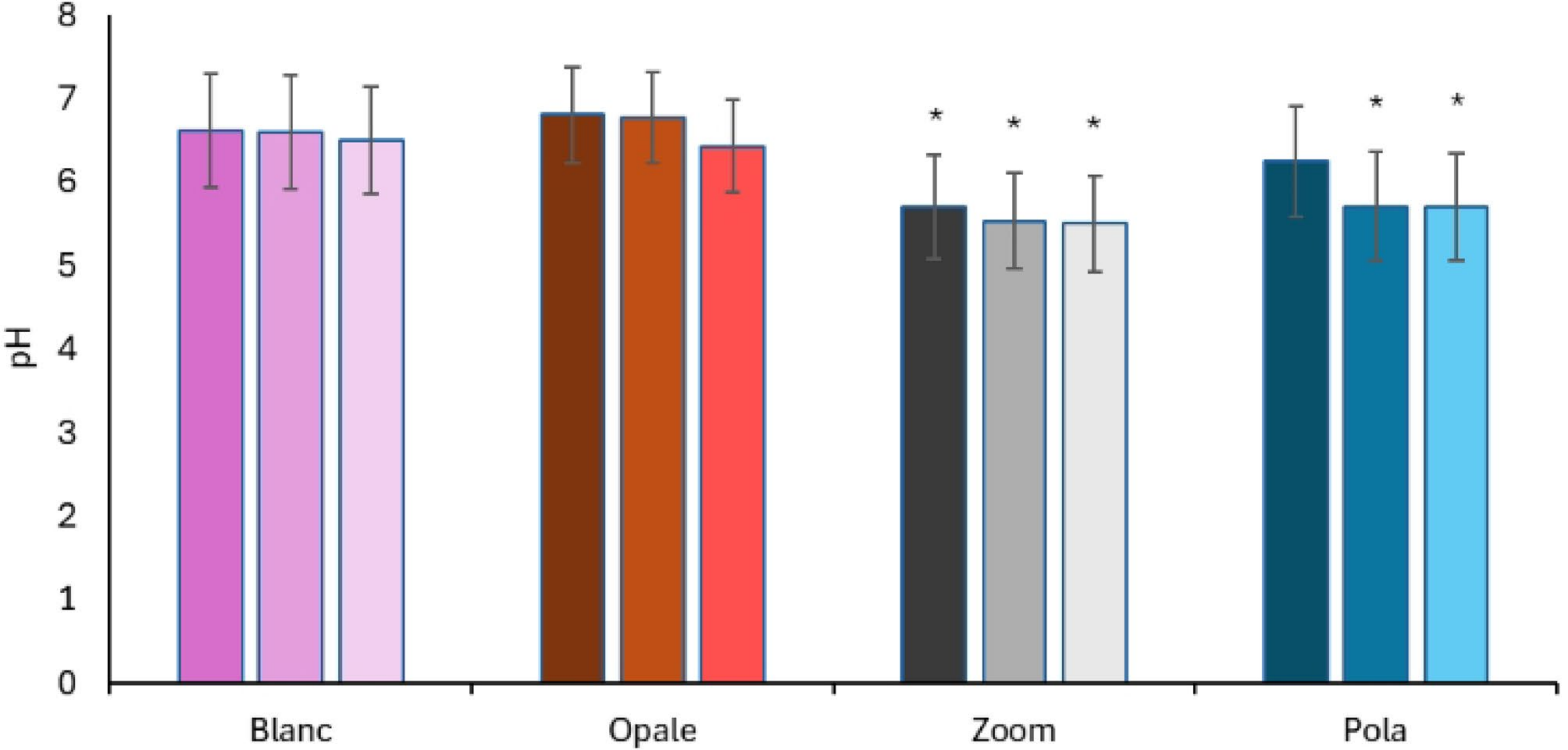
Measure the pH value of the whitening agent before and after the treatment of bovine mandible teeth. For each whitening agent, the first column represents the pH value at the initial time point (pH T0), the second after 4 h of treatment (pH T4), and the third after 8 h (pH T8). The x-axis labels represent the treatment groups. The symbol * indicates a significant difference (*p* < 0.05) compared to the values measured for Blanc and Opale.

The bleaching agents showed a marked tendency to a more acidic pH than at the start of the treatment (Fig. 9). However, the differences within each agent group did not appear statistically significant. Blanc and Opale had a less acidic pH than Zoom and Pola, which seemed to be substantial when tested statistically (*P* < 0.05).

## Discussion

In our study, we employed an ex vivo approach using bovine mandibular teeth to assess the efficacy of the analyzed at-home whitening treatments. While human teeth are not used, bovine teeth serve as ethical surrogates for human dental whitening studies, as they are derived from animals slaughtered for food production. Their reliability has been well-documented in the literature^13,34,35^. It has been demonstrated that bovine teeth share structural and chemical similarities with human teeth^36,37^. A recent systematic review and meta-analysis of in vitro studies by Soares et al. concluded that bovine teeth are a reliable substitute for human teeth in studies focusing on enamel and dentin substrates^38^.

De Dios Teruel et al.^37^ noted that both bovine and human enamel contain hydroxyapatite as the main component and exhibit comparable crystalline structures. However, the enamel prisms in bovine teeth differ slightly in orientation and size compared to those in human teeth, which could influence the light refractive and scattering dynamics post-whitening. Additionally, the porosity of bovine enamel enables simulation of absorption and interaction with whitening agents. However, bovine enamel is significantly thicker and has a higher organic matrix than human enamel^36^. From 2021, external bleaching tests on bovine teeth are validated by ISO 28.399:2021^22^.

While present, these differences do not compromise the model’s validity, as demonstrated by Hanna et al.^39^. Indeed, through a clinical case series with an 8-month follow-up on the BlancOne ULTRA + treatment with 35% HP^39^, they confirmed the results obtained in our ex vivo study using bovine mandibular teeth^16^. An interesting aspect of our methodology was the use of teeth from animals raised on the same organic farm, fed a uniform diet, and never given antibiotics before slaughter. This approach ensured consistency in the structure and initial brightness of the samples. Such uniformity is particularly significant compared to a previous study^16^, which found no statistically significant differences in initial brightness assessed via spectrophotometry.

Blanc and Opale were the most effective treatments analyzed, while Pola showed the lowest but still significant efficacy. Comparing at-home products with their professional counterparts of the same brands studied in a previous work^16^, it appears that, except for the BlancOne ULTRA + with 35% hydrogen peroxide (*P* < 0.05), the professional products Opalescence Extraboost (40% HP), Zoom WhiteSpeed (25% HP), and Pola Office (35% HP) did not demonstrate significantly superior efficacy compared to their at-home versions Opale, Zoom, and Pola (*P* > 0.05). This result aligns with a recent umbrella review by Aidos^40^, highlighting the absence of significant differences in color between in-office and at-home whitening techniques. Da Costa et al.^41^ suggested similar conclusions, indicating that multi-day at-home whitening produces results equivalent to single in-office treatment.

Interestingly, the effects of the at-home Blanc and Opale treatments were superior to those of Zoom and Pola’s professional treatments (*P* < 0.05)^16^. All the whitening treatments analyzed were effective, and the choice between one technique and another seems to depend more on factors such as cost, safety, and the time required rather than a significant difference in benefits. The results are consistent with a recent systematic review comparing different types of tooth whitening^42^.

When evaluating the safety of whitening products, pH and exposure time are crucial factors for enamel demineralization^43^. It is well-known that pH values below 5.5 and prolonged exposure times can dissolve hydroxyapatite crystals, contributing to enamel erosion^43,44^. However, the erosive effect also depends on saliva’s buffering capacity and protective ions, such as calcium and phosphate^45^.

Monitoring the pH of the tested products showed that Blanc and Opale had almost neutral pH values (6 < pH < 7), unlike Zoom and Pola, which had pH values around 5.5. The closeness of the former to neutrality could explain the higher efficacy, particularly for Blanc with 12% CP (corresponding to 4.2% HP), due to the greater stability of the CP and the gradual and effective release of the HP in the neutral or slightly alkaline pH range^46,47^. In terms of safety, all the products analysed were within ranges considered safe. No direct correlation was found between the products’ pH and adverse effects in the bovine teeth used in the study. However, it is likely that the formulation characteristics of whitening gels—such as viscosity, excipient composition, and the inclusion of functional additives—may play a critical role in modulating the balance between bleaching efficacy and enamel safety, as supported by evidence in the literature^48–50^.

In fact, enamel integrity analyses conducted using AFM, nanoindentation, and Young’s modulus analysis revealed significant differences in how the treatments alter the enamel’s physical properties, with important implications for safety and long-term use.

In particular, the AFM analysis demonstrated that Blanc and Pola had minimal impact on enamel roughness, preserving the tooth surface. In contrast, Zoom caused a moderate increase in roughness, suggesting mild demineralization or abrasive effects. On the other hand, Opale led to a significant increase in roughness, indicating aggressive enamel erosion. While the lower percentage of CP and the presence of nano-hydroxyapatite may support Blanc’s data, the shared percentage of both CP and potassium fluoride in Pola and Opale does not immediately explain the effects of these two distinctly different bleaching agents on enamel. It should be noted, however, that the protective effect of fluoride on enamel is markedly enhanced under acidic conditions, primarily due to the precipitation of calcium fluoride (CaF₂) and the formation of acid-resistant mineral phases such as fluorapatite^51–57^. Compounds such as stannous fluoride and titanium tetrafluoride demonstrate superior efficacy in these environments, whereas sodium fluoride shows comparatively limited surface interaction at neutral pH. Under neutral or alkaline conditions, no significant fluoride precipitation occurs, and the protective effect is mainly attributed to ionic fluoride promoting remineralization rather than surface adsorption^58–60^.

Referring to studies evaluating the influence of gel viscosity on whitening efficacy and enamel safety^61,62^, it appears that while Blanc falls within the medium–low viscosity range, the other gels (Opale, Pola, and Zoom) are indicated as high-viscosity formulations. In contrast, density values are comparable across all products (≈ 1.1 g/cm^3^). An increase in gel viscosity has been associated with a reduction in colour change, peroxide permeation, enamel demineralisation, and the release of reactive oxygen species (ROS), while simultaneously promoting a higher rate of peroxide decomposition during treatment^61^.

Accordingly, the pronounced reduction in enamel hardness and Young’s modulus observed with Opale, compared with Blanc, Pola, and Zoom, reflects the combined influence of formulation-specific factors. Although Opale, Pola, and Zoom share a similar carbamide peroxide content (16%, corresponding to 5.7% HP) and potassium fluoride, Opale lacks additional remineralising agents, such as amorphous calcium phosphate, which is present in Zoom. This absence may reduce enamel resilience to oxidative stress, thereby increasing its susceptibility to structural weakening^63^.

With a nearly neutral pH (6–7) and high viscosity, Opale exhibits more pronounced effects, likely due to enhanced gel retention and surface adhesion, which prolong local exposure to hydrogen peroxide and consequently intensify enamel damage^61,62^. Conversely, Pola, despite having comparable viscosity and peroxide concentration but a slightly acidic pH (~ 5.5), induces minimal alterations, suggesting that, also in our work, the chemistry of additives and pH play a key role in modulating peroxide penetration and mineral preservation^64^.

These findings indicate that enamel degradation is not solely determined by peroxide or fluoride content, but rather by the interplay among additive composition, the rheological properties of the gel matrix, and pH, which collectively regulate the balance between whitening efficacy and structural preservation.

From a long-term perspective, it should be emphasised that the level of degradation observed for Opale could compromise the protective function of enamel against mechanical forces and acid challenges, enhance bacterial adhesion, and promote secondary demineralisation, as previously reported^35,45^. Finally, when comparing these safety data for the at-home products with those of professional formulations analysed in our previous study^16^, Opale and Zoom demonstrated the most significant impact on dental enamel, whereas Blanc and Pola exhibited higher safety margins.

## Conclusions

All tested at-home whitening treatments demonstrated a measurable whitening effect after application, confirming their general efficacy. Among them, BlancOne Home Night + (12% CP with nano-hydroxyapatite) showed the best performance in balancing whitening efficacy and enamel preservation. While Opale also exhibited high bleaching effectiveness, it was associated with a more pronounced alteration of enamel surface properties, raising concerns regarding its long-term safety. These results highlight that the efficacy and safety of whitening treatments depend not only on the peroxide concentration but also on the complete formulation. Further in vivo studies are needed to evaluate potential effects on dentinal and gingival sensitivity.

## Data Availability

The data that support the findings of this study are available from the corresponding author upon reasonable request.
